# Complications are reduced with a protocol to standardize timing of fixation based on response to resuscitation

**DOI:** 10.1186/s13018-015-0298-1

**Published:** 2015-10-01

**Authors:** Heather A. Vallier, Timothy A. Moore, John J. Como, Patricia A. Wilczewski, Michael P. Steinmetz, Karl G. Wagner, Charles E. Smith, Xiao-Feng Wang, Andrea J. Dolenc

**Affiliations:** Department of Orthopaedic Surgery, MetroHealth Medical Center affiliated with Case Western Reserve University, 2500 MetroHealth Drive, Cleveland, OH 44109 USA; Departments of Orthopaedic Surgery and Neurosciences, MetroHealth Medical Center affiliated with Case Western Reserve University, 2500 MetroHealth Drive, Cleveland, OH USA; Division of Trauma, Department of Surgery, MetroHealth Medical Center affiliated with Case Western Reserve University, Cleveland, OH USA; Department of Neurosciences, MetroHealth Medical Center affiliated with Case Western Reserve University, Cleveland, OH USA; Department of Anesthesiology, MetroHealth Medical Center affiliated with Case Western Reserve University, Cleveland, OH USA

**Keywords:** Polytrauma, Fixation timing, Damage control, Femur fracture, Pelvis fracture, Spine fracture, Resuscitation

## Abstract

**Background:**

Our group developed a protocol, entitled Early Appropriate Care (EAC), to determine timing of definitive fracture fixation based on presence and severity of metabolic acidosis. We hypothesized that utilization of EAC would result in fewer complications than a historical cohort and that EAC patients with definitive fixation within 36 h would have fewer complications than those treated at a later time.

**Methods:**

Three hundred thirty-five patients with mean age 39.2 years and mean Injury Severity Score (ISS) 26.9 and 380 fractures of the femur (*n* = 173), pelvic ring (*n* = 71), acetabulum (*n* = 57), and/or spine (*n* = 79) were prospectively evaluated. The EAC protocol recommended definitive fixation within 36 h if lactate <4.0 mmol/L, pH ≥7.25, or base excess (BE) ≥−5.5 mmol/L. Complications including infections, sepsis, DVT, organ failure, pneumonia, acute respiratory distress syndrome (ARDS), and pulmonary embolism (PE) were identified and compared for early and delayed patients and with a historical cohort.

**Results:**

All 335 patients achieved the desired level of resuscitation within 36 h of injury. Two hundred sixty-nine (80 %) were treated within 36 h, and 66 had protocol violations, treated on a delayed basis, due to surgeon choice in 71 %. Complications occurred in 16.3 % of patients fixed within 36 h and in 33.3 % of delayed patients (*p* = 0.0009). Hospital and ICU stays were shorter in the early group: 9.5 versus 17.3 days and 4.4 versus 11.6 days, respectively, both *p* < 0.0001. This group of patients when compared with a historical cohort of 1443 similar patients with 1745 fractures had fewer complications (16.3 versus 22.1 %, *p* = 0.017) and shorter length of stay (LOS) (*p* = 0.018).

**Conclusions:**

Our EAC protocol recommends definitive fixation within 36 h in resuscitated patients. Early fixation was associated with fewer complications and shorter LOS. The EAC recommendations are safe and effective for the majority of severely injured patients with mechanically unstable femur, pelvis, acetabular, or spine fractures requiring fixation.

## Background

Early stabilization of femur, pelvis, acetabulum, and spine fractures minimizes pulmonary and other complications, while damage control tactics may provide provisional stability in patients too unstable to tolerate definitive surgery [1–18]. Currently, controversy exists regarding indications for damage control procedures [11–13, 19, 20]. Unnecessary delay of definitive care has been associated with pulmonary complications, deep venous thrombosis, skin breakdown, and sepsis [2, 4–6, 18, 21–23]. Delay is also associated with longer hospital stays and ventilation times [1, 2, 10, 14, 17, 18, 24]. Ultimately, delay can generate increased costs of care and opportunity costs in terms of limited access for other patients to a given hospital system [25].

We developed a protocol to determine timing of definitive fracture care based on the presence and severity of metabolic acidosis as measured by arterial pH or base excess and/or venous lactate [26]. Key features of this protocol are simplicity, ease of assessment, universal application to all patients, and agreement among all trauma providers in the implementation thereof. The purpose of this project was to prospectively assess the safety and feasibility of this protocol, defined as Early Appropriate Care (EAC). We hypothesized that (1) utilization of EAC would be associated with fewer complications than a historical cohort of similar patients and that (2) EAC patients treated definitively for their fractures of interest within 36 h of injury would have fewer complications than those who had protocol violations and treated on a delayed basis.

## Methods

Three hundred thirty-five consecutive, skeletally mature patients with Injury Severity Score (ISS) ≥16 and 380 fractures of the proximal or diaphyseal femur (*n* = 173), pelvic ring (*n* = 71), acetabulum (*n* = 57), and/or spine (*n* = 79) were treated surgically over 30 months. Associated injuries of the chest (*n* = 209), abdomen (*n* = 97), and head (*n* = 192) were present. When the Abbreviated Injury Score (AIS) for a region was ≤2, the injuries were defined as minor, while AIS >2, were considered severe. Fracture characteristics, associated injuries, medical co-morbidities, and the timing and techniques of provisional treatment and surgical procedures were documented.

### EAC protocol

Inclusion criteria were patients with mechanically unstable fractures of the proximal or diaphyseal femur, pelvic ring, acetabulum, and/or spine requiring fixation. All patients had a presenting ISS ≥16 and at least one of the following: injury to one or more other body systems, hemodynamic instability on presentation, defined by hypotension (systolic blood pressure <90), tachycardia (heart rate greater than 110), and/or immediate transfusion requirements. Exclusion criteria included fractures sustained from a low energy mechanism or secondary to neoplasm and those in skeletally immature patients.

The EAC protocol recommended definitive fixation of the fractures of interest within 36 h of injury, as long as initial acidosis had improved to at least one of the following: lactate <4.0 mmol/L, pH ≥7.25, or base excess (BE) ≥−5.5 mmol/L. The protocol parameters were developed via a model previously reported [26]. All patients were also required to be responding to resuscitation without pressor support. Venous lactate is routinely obtained at the time of presentation to our facility. Arterial blood gas measurements were obtained at the discretion of the treating general trauma surgeon. Labs were repeated a minimum of every 8 h until normal, both preoperatively and postoperatively.

In cases of persistent acidosis, damage control was recommended if fractures were amenable to this. Ongoing clinical and laboratory reassessment was performed, and a safe time for definitive fixation was determined by the attending trauma surgeon. In the event of ongoing active bleeding secondary to fracture, with failure to respond to conventional methods including splinting, transfusion, and angiography, the general trauma surgeon could recommend fixation or amputation as a life saving measure. For patients with two or more of the fractures of interest, the orthopedic trauma surgeon would propose a plan for sequence of fixation. The general trauma surgeon would review this plan and propose incorporation of any other procedures warranted prior to fracture care or within the same surgical setting. The trauma surgeon, in conjunction with the orthopedic trauma and anesthesia teams, would reassess the patient during the first of multiple procedures and sequentially thereafter to determine the safety of continuing surgery. Worsening acidosis would be considered an indication to delay additional procedures. Notably, some patients would have open extremity fractures for which a minimum of urgent debridement and irrigation followed by splinting and/or external fixation was anticipated in the initial surgical setting. Standard inpatient protocols for antibiotic usage, DVT prophylaxis, and nutrition were in place and unchanged throughout the study period.

### Measurements of outcome

Complications included infections, sepsis, DVT, organ failure, and pulmonary complications: pneumonia, acute respiratory distress syndrome (ARDS), and pulmonary embolism (PE). Sepsis was defined by positive blood culture and at least two of the following: temperature >38 °C or <36 °C, heart rate >90 beats/minute, respiratory rate >20 breaths/min, and white blood cell count >12,000/mL, <4,000/mL, or >10 % band forms [27]. Deep vein thrombosis proximal to the knee was diagnosed on duplex ultrasound, and PE was diagnosed on computed tomography. Acute renal failure was defined as a 50 % increase in creatinine from baseline [28]. Multiple organ failure was defined as two or more organs in failure for a minimum of three consecutive days with a score of four or more points [29]. Acute respiratory distress syndrome was defined as PaO_2_/FiO_2_ ratio of less than 200 for more than four consecutive days with diffuse infiltrates on chest radiographs, in the absence of pneumonia [30]. Pneumonia was defined by quantitative culture obtained via bronchoscopy and bronchoalveolar lavage; decision to perform this procedure was made by the attending trauma intensivist and was generally prompted by new pulmonary infiltrate on plain chest radiograph in conjunction with purulent sputum, temperature greater than 38 °C and/or a white blood count greater than 10,000/mL [30].

An adjudication committee consisting of physicians from anesthesiology, orthopedic surgery, neurosurgery, and trauma surgery/surgical critical care independently reviewed all records at 6-month intervals. Adherence to the protocol was assessed, including reasons for delay of surgery. None of the adjudicating physicians participated in the collection or analysis of research data.

### Statistical analysis

Continuous variables were compared using the two sample *t* test, and categorical variables were compared using the Pearson’s chi-square test. The study group was divided into two groups based upon timing of fixation: before or after 36 h. For the binary outcome measures, logistic regression models were created. For length of stay (LOS) and ventilation times, generalized linear regression models with negative binominal distribution were used. This is due to the fact that days are non-negative integers and have a right-skewed distribution. Both univariate and multivariate logistic regression models and negative-binomial regression models were applied to find potential risk factors. Risks factors included patient age, gender, ISS, type of fracture, and type and severity of other system injuries, including those to the head, chest, and abdomen. All analyses were performed by a statistician not involved in the treatment of the patients. The SAS statistical package was used (Version 9.3 for Unix., SAS Inc., Cary, NC, USA). The level of statistical significance was set at *p* < 0.05 (two-tailed).

### Statement of ethical approval

Institutional Review Board approval was obtained from the MetroHealth Institutional Review Board prior to initiating this study: #IRB07-01157.

All devices used in this study are FDA-approved.

## Results

Three hundred thirty-five consecutive patients with 380 fractures were prospectively assessed and treated according to EAC criteria. This included 239 men and 96 women with mean age of 39.2 years and mean ISS of 26.9 (Table 1) and injuries to other systems (Table 2). Two hundred sixty-nine patients (80 %) had 301 fractures treated within 36 h of injury, according to the EAC protocol, and 66 (20 %) patients had 79 fractures fixed on a delayed basis (protocol violations), although all patients met the desired EAC resuscitation parameters, with improvement of acidosis, within 36 h of injury. Femoral fractures were more likely to be treated within 36 h (90 %, *p* = 0.0003), and spinal fractures were more likely to be treated later than 36 h from injury (34 %, *p* < 0.0001). Patients with spine fractures which were treated more than 36 h after injury received more blood transfusions versus those treated earlier (9.7 versus 4.3 U, *p* = 0.032); however, other fracture subtypes had no differences in the number of units of blood transfused when early and delayed groups were compared.

**Table 1:** Demographic information on EAC patients treated with definitive fixation within 36 h of injury versus those treated later

	All patients: 335 with 380 fractures	Definitive fixation ≤36 h: 269 patients with 301 fractures	Definitive fixation >36 h: 66 patients with 79 fractures	*p* value
Male	239 (71 %)	199 (74 %)	40 (61 %)	0.04
Female	96 (29 %)	70 (26 %)	26 (39 %)	
Mean age (years)	39.2	39.1	39.6	0.31
Range	14 to 91	14 to 91	17 to 82	
Mean ISS	26.9	25.1	34	0.0005
Range	16 to 66	16 to 66	16 to 66	
Mechanism of injury				
MVC	170 (51 %)	131 (49 %)	39 (59 %)	
MCC	52 (16 %)	47 (17 %)	5 (7.6 %)	
Fall from height	63 (19 %)	54 (20 %)	9 (14 %)	
Pedestrian vs MVC	20 (6.0 %)	14 (5.2 %)	6 (9.1 %)	
GSW	15 (4.5 %)	14 (5.2 %)	1 (1.5 %)	
Crush	8 (2.4 %)	4 (1.5 %)	4 (6.1 %)	
Other	7 (2.1 %)	5 (1.9 %)	2 (3.0 %)	
Fractures				
Femur	173 (52 %)	155 (58 %)	18 (27 %)	*p* = 0.0003
Pelvis	71 (21 %)	48 (18 %)	23 (35 %)	*p* = 0.07
Acetabulum	57 (17 %)	46 (17 %)	11 (17 %)	*p* = 0.39
Spine	79 (24 %)	52 (19 %)	27 (41 %)	*p* < 0.0001
Patients with 1 fracture	291 (87 %)	236 (88 %)	55 (83 %)	*p* = 0.35
Patients with 2 fractures	40 (12 %)	31 (12 %)	9 (14 %)	
Patients with 3 fractures	3 (0.9 %)	1 (0.4 %)	2 (3.0 %)	

*ISS* Injury Severity Score, *MVC* motor vehicle collision, *MCC* motorcycle crash, *GSW* gunshot wound

Percentages of all patients in each column with a given gender, mechanism, and type of fracture are shown in parentheses

**Table 2:** Associated injuries. The numbers of patients with associated abdominal, chest, and/or head injuries are listed

	Fixation within 36 h (*n* = 269)	Fixation after 36 h (*n* = 66)	Totals (*n* = 335)	
Abdominal injury	66 (25 %)	31 (47 %)	97 (29 %)	*p* = 0.0002
Minor (AIS ≤2)	37 (14 %)	12 (18 %)	49 (15 %)	NS
Severe (AIS >2)	29 (11 %)	19 (29 %)	48 (14 %)	*p* < 0.0001
Chest injury	167 (62 %)	42 (64 %)	209 (62 %)	NS
Minor (AIS ≤2)	75 (28 %)	13 (20 %)	88 (26 %)	NS
Severe (AIS > 2)	92 (34 %)	29 (44 %)	121 (36 %)	*p* = 0.14
Head injury	149 (55 %)	43 (65 %)	192 (57 %)	*p* = 0.08
(GCS 9–15)	109 (41 %)	26 (39 %)	135 (40 %)	NS
(GCS ≤8)	40 (15 %)	17 (26 %)	57 (17 %)	*p* = 0.018

*P* values are listed for comparisons of the groups treated within 36 h of injury versus later

*AIS* Abbreviated Injury Score, *GCS* Glasgow Coma Scale

Reasons for treatment after 36 h following injury are shown in Table 3. Surgeon choice to delay was the most common reason, occurring in 71 %. An operating room was not available in four other cases (6.1 %). Five patients were determined to be medically unstable: two requiring pressor support for hypotension, two with concern for myocardial infarction, and one with severe hyperkalemia. Two patients were treated on a delayed basis due to traumatic brain injury with severely elevated intracranial pressures (3.0 %).

**Table 3:** Reasons for surgical delay in patients treated definitively more than 36 h after injury. Sixty-six patients had definitive surgery for 79 fractures on a delayed basis. EAC resuscitation criteria were met in all cases within 36 h

Reason for delay	Number of patients (%)
Surgeon choice	47 (71 %)
Intensivist choice	6 (9.1 %)
Medically unstable	5 (7.6 %)
Operating room unavailable	4 (6.1 %)
Severe head injury	2 (3.0 %)
Patient choice	2 (3.0 %)

Three patients (0.9 %) had protocol violations due to definitive fixation prior to meeting the resuscitative criteria. They were included with the patients treated definitively within 36 h in the final analysis. One other patient underwent damage control external fixation of a femur shaft fracture due to persistent acidosis and elevated intracranial pressure. Her systemic status never improved, and she died from her head injury 4 days later. She never received definitive fixation; thus, she was excluded from further analysis.

Complications occurred in 16.3 % of patients treated within 36 h and in 33.3 % of delayed patients (*p* = 0.0009), including pneumonia in 8.2 and 13.6 % (*p* = 0.09, Table 4), respectively. Multivariate logistic regression accounted for potential confounders including age, gender, ISS, type of fracture, and type and severity of other system injuries, and patients with fixation within 36 h had a lower total complication rate (*p* = 0.0058). Greater age was also associated with a higher complication rate after multivariate analyses (*p* = 0.0074). The delayed group had more sepsis (18.2 versus 2.2 %, *p* < 0.0001) when compared to patients treated within 36 h. Delayed surgery was also associated with acute renal failure (7.6 versus 0.4 %, *p* = 0.0001). Death occurred overall in 1.5 % of patients at mean 21.2 days. Causes included: respiratory failure in two, sepsis in one, and organ failure in two. Two patients died after hospital discharge (5 and 7 months) of unknown causes. Deep venous thrombosis occurred in 1.8 % of patients overall; all were patients in the early fixation group.

**Table 4:** Complications in EAC patients treated within 36 h versus later. Comparison is made with a previously described cohort of patients with the same types of fractures managed at the same trauma center

	All EAC patients (*n* = 335)	EAC treated ≤ 36 h (*n* = 269)	EAC treated >36 h (*n* = 66)	Historical comparison group (*n* = 1443)
Any complication	66 patients (19.7 %) with 90 complications	44 patients (16.3 %) with 57 complications	22 patients (33.3 %) with 32 complications*	319 patients (22.1 %) with 354 complications
Pneumonia	31 (9.3 %)	22 (8.2 %)	9 (14 %)	118 (8.2 %)
DVT	6 (1.8 %)	6 (2.2 %)	0	92 (6.4 %)
PE	8 (2.4 %)	7 (2.6 %)	1 (1.5 %)	22 (1.5 %)
ARDS	5 (1.5 %)	4 (1.5 %)	1 (1.5 %)	33 (2.3 %)
ARF	6 (1.8 %)	1 (0.37 %)	5 (7.6 %)*	24 (1.7 %)
MOF	2 (0.60 %)	1 (0.37 %)	1 (1.5 %)	5 (0.35 %)
Infection	9 (2.7 %)	6 (2.2 %)	3 (4.5 %)*	12 (0.83 %)
Sepsis	18 (5.4 %)	6 (2.2 %)	12 (18 %)*	33 (2.3 %)
Death	5 (1.5 %)	4 (1.5 %)	1 (1.5 %)	15 (1.0 %)

*Denotes statistical significance versus patients treated within 36 h, *p* < 0.01 in all cases

Sixty-two percent (209 of 335) of our patients had a chest injury; 88 (42.1 % of 209) were minor injuries and 121 (57.9 % of 209) were severe. Mean time from injury to fixation was no different when patients with and without chest injury were compared (29.2 versus 25.5 h; *p* = 0.53). However, patients with chest injuries were significantly more likely to have pulmonary complications (17.2 %, *p* = 0.001) and any complication (25.8 %, *p* = 0.0005). Eighty-three percent of the pulmonary complications in chest-injured patients occurred in those with severe injuries.

A separate analysis was undertaken to evaluate patients *without* chest injury (*n* = 126). When fixation was within 36 h of injury, 15 % had complications versus 28 % with later fixation (*p* = 0.15). Shorter ventilation times and ICU and total hospital stays were noted with early fixation (all *p* < 0.007). When patients with no chest injury or chest injury AIS ≤2 (*n* = 88) were combined, significantly fewer complications were noted with early fixation (*p* = 0.004), in addition to shorter hospital stays and ventilation times (all *p* < 0.001).

Seventy-nine patients were treated surgically for spine fractures. Thirty-one had spinal cord injury (39 %), with equal distributions in the early and delayed groups. Chest injury occurred in 59 % of the spine fracture patients. Despite an equal proportion of chest-injured patients, with similar injury severity, complications occurred more frequently in patients with spine fractures versus other fractures (28 %, *p* = 0.01, Table 5). Early definitive spine fixation did not increase complications; in contrast, the mean time to fixation of spine fractures in patients without complications was 26 versus 68 h in patients who developed complications (*p* < 0.0001). Surgeon choice was the reason for delayed fixation in 61 % of cases.

**Table 5:** Complications in patients treated surgically for spine fractures versus patients treated for fractures of the pelvis, acetabulum, and/or femur

	Thoracolumbar spine fracture	Other fractures
Fixation ≤36 h (*n* = 269)	52 patients	217 patients
Any complication	13 (25 %)*	31 (14 %)
Pulmonary complication	11 (21 %)*	18 (8.3 %)
Fixation >36 h (*n* = 66)	27 patients	39 patients
Any complication	9 (33 %)**	13 (33 %)***
Pulmonary complication	5 (19 %)	2 (5.1 %)
All patients (*n* = 335)	79 patients	256 patients
Any complication	22 (28 %)*	44 (16 %)
Pulmonary complication	16 (20 %)*	20 (7.8 %)

*all *p* < 0.03 for patients with spine fractures versus other fractures

**p = 0.04 for spine patients treated within 36 h versus later

***p = 0.002 for patients without spine fractures treated within 36 h versus later

Additional analysis was performed to review patients who were not treated for spine fractures and to compare them with the thoracolumbar spine patients (Table 5). Mean age was 39 years for both groups, and mean ISS was 27.0 for spine patients and 26.8 for the other patients. Complications occurred in 33 % in both groups when fixation was undertaken more than 36 h after injury. Similar to spine patients, fixation performed within 36 h of injury was associated with fewer complications in patients treated for fractures of the pelvis, acetabulum, and/or femur (14 versus 33 %, *p* = 0.002). Notably, patients treated for femoral shaft fractures incurred fewer complications than patients treated for other fracture types (6.9 % overall and 5.8 % when treated within 36 h, both *p* < 0.0001).

This entire group of EAC patients was compared with a historical cohort of 1443 similar patients with 1745 fractures treated at the same hospital between 2000 and 2007 (Table 4). A propensity score matching approach was used to account for confounding variables, including age, gender, and associated injuries. Pulmonary complications were noted in 12.8 % of the matched group versus 10.7 % of EAC patients, *p* = 0.15. Complications occurred in 19.7 % of all EAC patients versus 22.1 % in the historical group, *p* = 0.17. When comparing the EAC patients treated within 36 h with the matched historical cohort, fewer total complications (16.3 versus 22.1 %, *p* = 0.017) were noted with EAC. In comparing the patients treated within 36 h with the EAC protocol versus our historical group, pulmonary complications were noted in 10.8 versus 17.8 %, *p* = 0.09, and significantly shorter ICU (*p* = 0.048) and hospital stays (*p* = 0.018) were seen for EAC patients.

Mean LOS was 10.8 days, with mean of 6.01 ICU days (Table 6). Both were significantly shorter in patients treated definitively within 36 h of injury: 4.35 versus 11.6 days and 9.52 versus 17.3 days (both *p* < 0.0001). Negative-binomial regression models were applied to account for potential confounders, and all differences remained significant. Time of mechanical ventilation was also significantly shorter in the early group.

**Table 6:** Hospital length of stay (LOS), ICU stay, and ventilation times for EAC patients treated within 36 h of injury versus later. Data are presented as mean and standard deviation

	All EAC patients (*n* = 335)	EAC ≤36 h (*n* = 269)	EAC >36 h (*n* = 66)	*p* value
ICU LOS (days)	6.01 ± 7.8	4.35 ± 7.0	11.6 ± 8.6	<0.0001
Total LOS (days)	10.8 ± 8.1	9.52 ± 7.8	17.3 ± 9.1	<0.0001
Ventilation time (days)	3.75 ± 6.6	2.57 ± 5.7	7.6 ± 7.0	<0.0001

## Discussion

Early definitive stabilization of femoral shaft fractures and other major skeletal injuries was advocated by various surgeons over 30 years ago [2, 5, 21]. Higher incidences of pneumonia, ARDS, fat embolism, longer hospital stays, and higher costs were associated with delayed fixation, and benefits of early fixation were more profound in patients with multiple system trauma versus isolated injury. Soon, early *total* care became common, including the fixation of all fractures in the initial surgical setting.

Occasional development of severe complications led some to question early total care for all patients [31–33]. Pape, et al. suggested femoral nailing in patients with lung contusion may cause ARDS. Although early nailing was beneficial in patients without chest injury, 33 % with severe chest injury developed ARDS after early nailing [34]. Fractures cause pain and hemorrhage, and fixation of fractures relieves some of this. However, bleeding and inflammation related to the surgery itself must be considered. This “second hit” in an inadequately resuscitated patient can be associated with deleterious systemic inflammation and organ dysfunction [1, 30, 33].

Damage control orthopedics (DCO) minimizes surgical duration and bleeding and is an alternative in critically ill patients [10, 11, 13, 16, 19, 32]. Guidelines to stratify patients into stable, borderline, unstable, and in extremis groups based on types of injury to all systems and physiological and laboratory parameters have been proposed, but not validated, suggesting DCO for unstable and in extremis categories [32]. Appropriate patient selection for DCO would be helpful in minimizing the additional costs associated with a second procedure. Furthermore, some femur fractures are not manageable with external fixation. This issue also applies to many pelvic ring, spine, and acetabulum fractures. This group of injuries constitutes a similar set of clinical issues for a trauma patient, in that all of them generate pain and require bedrest and recumbency until they are stabilized. Although the majority of prior literature regarding early fracture care has focused on femoral shaft fractures, benefits of early stabilization of pelvis [4, 6, 19, 24, 35], acetabulum [14, 17], and spinal fractures have also been described [8, 36–41]. We proposed that one algorithm for patient risk assessment could be developed to address these common trauma scenarios, going beyond treating only the femur [26].

Utility of any treatment algorithm is based on several crucial features. An algorithm must be simple to remember and easy to apply. It should be associated with consistent results or outcomes independent of the individuals involved in the decision-making. Ideally, it would be applicable to a wide group of patients without a change in risk. The EAC protocol was developed by a multi-disciplinary group of physicians, keeping these features in mind. The laboratory parameters are routinely obtained, rapidly available, and low in cost. Serial measurements of venous lactate are more specific than BE or pH [42–44], but each of these measurements shows response to resuscitation [45–48]. Over the initial 30 months, protocol violations occurred in only three patients (0.9 %) for surgery without meeting resuscitative criteria and in 66 resuscitated patients (20 %) for delayed surgery.

Eighty percent of EAC patients were treated within 36 h, as recommended. Femur fractures were most likely to be treated within the recommended time, and spine fractures were most likely to be managed on a delayed basis, consistent with historical practice patterns. Surgeon choice and other nonmedical and operational issues accounted for 89 % of the patients who were delayed. Most often, this was due to preferences to do these cases during regular weekday block time. Patients treated on a delayed basis had higher frequency of both severe head injury and severe abdominal injury; thus, although they met the resuscitative criteria according to laboratory parameters, surgeons may have been more inclined to treat them on a delayed basis. Our analysis attempted to account for these differences through multivariate regression. It is possible that other patient factors, including coagulopathy may have influenced surgeon decision to delay definitive care. Over the 20 months of the study, fewer nonmedical delays occurred. Currently, less than 10 % of our resuscitated patients undergo fixation after 36 h.

The EAC protocol was developed with a system goal to optimize care of multiply-injured patients. The algorithm was based on a predicted reduction of pulmonary and other complications. Prior modeling showed fewer complications when resuscitated patients were treated within 24 h and to a lesser extent within 48 h [26]. Thus, 36 h was chosen as a realistic time frame for adequate resuscitation and for surgeons and operating rooms to be available during daytime hours. We anticipated most patients from other facilities would also arrive within several hours and could be optimized for surgery within the desired timeframe. To date, all patients have met the recommended parameters within 36 h.

When comparing EAC patients who underwent definitive fixation within 36 h versus the historical group, fewer complications were noted with EAC. Enhancements in critical care and ventilation strategies may have contributed, as the historical group was treated between 2000 and 2007. Our study was underpowered to detect a difference in the rates of pulmonary complications between the EAC and historical groups. With a 20 % rate of nonadherence to the protocol, we would have needed to study 419 patients.

We also hypothesized that EAC patients treated definitively within 36 h of injury would have fewer complications versus on a delayed basis. Complications were over twice as frequent in patients with delay; sepsis and ARF were significantly more common, but our study is likely underpowered to detect differences in survival. Significantly higher rates of sepsis have been previously reported with delayed fixation. This is believed to be secondary to increased gut permeability to bacterial translocation. Mechanical ventilation, which was significantly prolonged with delayed fixation, has also been shown to increase the tendency for bacterial translocation and infection. Length of stay was also shorter when definitive fixation was undertaken within 36 h, consistent with prior literature which has shown fewer complications and shorter LOS with early surgery for fractures of the femur [2, 3, 7, 10, 12, 15, 22, 49, 50], pelvis [4, 6, 17, 24], acetabulum [14, 17], or spine [8, 36–41, 51].

O’Toole et al. reviewed patients treated with early intramedullary femoral nailing with lactate <2.5 mmol/L and noted acceptably low rates of ARDS when compared with more critically ill patients managed with DCO (1.5 % versus 0, NS) [12]. They concluded that with attention to adequate resuscitation, early primary intramedullary nailing is safe in multiple trauma patients. Our patients also developed ARDS infrequently.

Other authors have shown low complication rates when patients were stratified into borderline, unstable, and in extremis groups to determine indications for DCO [19, 32]. We agree in concept with including physiological or laboratory parameters that reflect adequacy of resuscitation. However, we disagree in relegating patients to unstable or other categories, and delayed definitive fixation, purely because they have a severe type of chest, pelvis, and/or extremity injury. It is the hemorrhage associated with some injuries that is problematic, and this bleeding would be reflected in vital signs and laboratory parameters. Furthermore, presence of severe chest injury is a risk factor for pulmonary complications, independent of timing of fixation. Several prior reports have shown that patients with severe chest injury are more likely to develop pulmonary complications when subjected to delayed fixation [10, 11, 17, 21, 51, 52]. Our study supports this, in minimizing complications when resuscitated patients are managed definitively on an early basis.

The strengths of this study include a large sample. All patients were prospectively assessed and treated with this algorithm. One possible criticism is that the threshold for adequacy of resuscitation is too high, potentially creating an unsafe situation for some. However, this algorithm was based on probability modeling in a very large sample, suggesting its safety [26]. The EAC parameters demonstrated reductions in complications when compared with our historical practice, and our results compare favorably with other published data [7, 9, 11, 15, 24, 27, 50].

Another potential criticism of EAC could be failure to adjust threshold parameters based on type of fracture and anticipated surgical duration and hemorrhage. Probst et al. reviewed 213 patients with pelvic ring fractures and concluded that surgery in excess of 3 h was more often associated with liver failure, while delayed surgery (days 4 to 7) may reduce mortality unless surgical duration is under 1 hour, in which case early surgery was beneficial [53]. We agree that longer procedures carry more surgical risk, in terms of infectious and thrombotic complications. However, our data suggest that in an adequately resuscitated patient, earlier definitive surgery appears acceptable with fewer complications than when the same surgery is delayed. We believe that fracture stabilization is part of the resuscitation, as it reduces pain and bleeding. Our algorithm encourages continuous reassessment during procedures to ensure that resuscitation is occurring, in order to account for bleeding generated from the surgery itself.

Several limitations to the current EAC protocol were noted by our team over the course of study. Patients of advanced age or with medical risk factors may require additional cardiac or other diagnostic tests as part of their pre-operative risk assessment and optimization. This occurred in <1 % of our patients. Occasionally, patients have met the prescribed resuscitation criteria but were critically ill due to severe head injury with elevated intracranial pressure, evolving myocardial ischemia or infarct, or other medical reasons. Such issues required delay in 2 % of our patients.

## Conclusions

In summary, definitive stabilization of fractures of the femur, pelvis, spine, and acetabulum should occur when patients are adequately resuscitated to prevent a deleterious reactive systemic inflammatory response. Our protocol recommends definitive fixation within 36 h in resuscitated patients as defined by at least one of the following: venous lactate <4.0 mmol/L, BE ≥−5.5 mmol/L, or pH ≥7.25. All 335 patients had achieved the desired level of resuscitation within that time. Some were treated on a delayed basis for medical reasons, but surgeon choice and other nonmedical and operational issues accounted for 89 % of delays. Early fracture care resulted in fewer complications and shorter LOS in EAC patients, and EAC patients had fewer complications than our historical group, when controlling for age, timing of fixation, and severity of other injuries, suggesting improvement with a standardized protocol to assess adequacy of resuscitation. EAC recommendations appear safe.
